# Effects of hospice care on quality of life and negative emotions in patients with advanced tumor: A protocol for systematic review and meta-analysis

**DOI:** 10.1097/MD.0000000000020784

**Published:** 2020-07-02

**Authors:** Jing Fu, Yan Zeng, Yan Tan, Baiyu Fu, Haiyan Qiu

**Affiliations:** a Department of Gastroenterology, the First Affiliated Hospital of Hainan Medical University, Haikou; b Department of Emergency; c Department of Gastroenterology, Danzhou People's Hospital, Danzhou, Hainan, China.

**Keywords:** advanced tumor patients, hospice care, negative emotions, PRISMA-P, quality of life, randomized controlled trial, routine nursing

## Abstract

**Background:**

To evaluate the effect of hospice care on the quality of life and negative emotion of advanced tumor patients systematically, which compared with routine nursing mode, improving the quality of life of advanced tumor patients, reducing the negative emotion of advanced tumor patients, and providing evidence-based medicine for better implementation of targeted service of hospice care.

**Methods:**

Computer retrieval network electronic database: Retrieval CNKI, Chinese clinical trial registry, WANFANG database, China Biology Medicine disc, CQVIP database, PubMed, Embase, The Cochrane Library, and Web of Science database on the clinical research of hospice care on the quality of life and negative emotions of advanced tumor patients. While dating from the references included in the study, manually retrieving relevant tabloids, papers, and related journals without electronic version. The retrieval strategy adopts the combination of subject words and free words. The range of searching time was from the beginning of each database to April 1, 2020. According to the inclusion and exclusion criteria, the 2 researchers selected the literature and extracted the data independently, and used the Cochrane system evaluator manual 5.1.0 to conduct a bias risk assessment of the literature, which was finally included in the study. If two researchers disagree in the process of literature selection, a third researcher is invited to join in, discuss the issues that have differences, and then make a decision. RevMan 5.3.3 software and Stata 14.0 software were used to conduct the meta-analysis of the included research.

**Results:**

According to the process of Preferred Reporting Items for Systematic Review and Meta-Analysis Protocols (PRISMA-P), this study will be carried out strictly, and the results of research will be published publicly in high-quality international academic journals with peer review.

**Conclusion:**

Through the study, we will arrive at whether hospice care has advantages in improving the quality of life and negative emotion of advanced tumor patients, which compared with routine nursing mode, and the formulation of individualized hospice care strategy to provide the basis for the application of hospice care in the treatment of end stage tumor patients.

**Registration:**

OSF platform, registration number: 47enh.

## 1 Introduction

Malignant tumors, also known as cancer, are generally called a group of diseases, which affect any part of the body. In the past 30 years, the incidence rate and mortality rate of malignant tumors have shown a trend of increasing year by year, which has become an important public health problem that threatens people's life and health seriously. Between 1990 and 2013, the proportion of deaths caused by malignant tumors in the proportion of whole deaths, increased from 12% to 15%.^[1,2]^ According to the latest published statistics of the World Health Organization, it shows that there were 18.1 million new cases and 9.6 million death cases all over the world in 2018, of which 48.4% new cases and 57.3% death cases happened in Asian countries. The highest disease rate of cancer was lung cancer, breast cancer, prostate cancer, colorectal cancer, and the highest mortality rate was lung cancer, colorectal cancer, gastric cancer, and liver cancer.^[3–5]^ The incidence and death of malignant tumors in China is also extremely severe, the *2017 China Tumor Registration Annual Report* pointed out that China has 4.29 million new tumor cases per year, accounting for 20% of new cases all over the world, and reaches up to 2.81 million deaths cases per year.^[6]^ In other words, about 10,000 people are diagnosed as malignant tumors every day in China, with an average of 7 persons being diagnosed as malignant tumors per minute. In the future, the disease rate of tumor will increase at a rate of 3% to 5%.^[7]^

Currently, cancer is a major disease to threat the human health, most cancer patients’ early symptoms are not obvious, when it was found, it was in the late stage. Because of the limitation of medical level, there is no effective treatment for advanced cancer, and only though the existing medical means to maintain patient's life.^[8]^ Because of the double pressure of physical and mental caused by disease, most advanced cancer patients are negative and pessimistic. To improve the patients’ quality of life before the death, to alleviate the pain of patients, and to help patients more comfortably and calmly spend the last stage of life, is the pursuit of medical staff.^[9]^ Therefore, when patients with malignant tumors have developed distant or extensive metastasis, which is what we usually call the advanced tumor stage, the main anti-tumor treatment has no obvious therapeutic effect at this time, and palliative treatment is used as the main treatment of patients with end-stage cancer is used in clinical.^[10]^ When malignant tumors are at the end stage and expected lifetime is only for a few weeks to months, hospice services are often advocated. With the development of palliative medicine and hospice care, the psychological problems and psychological intervention of advanced malignant tumors patients have been paid more attention gradually.^[11]^

Hospice care is a new interdisciplinary subject. It is a health care service that provides physiological, psychological, social support and care to the patients and their families who are facing death and cannot be cured at the current medical level. It embodies the concept of holistic nursing and the spirit of medical humanism. The main purpose of hospice care is to provide scientific and reasonable nursing services when the patient is dying, so as to reduce the patient's physical pain and the negative emotions of the patient and his family. Respect the life of the dying patient and enable him to walk the final journey of his life without pain, tranquility and comfort.^[12,13]^ Hospice care for advanced tumors patients includes symptomatic treatment, nutritional support, functional exercise, and recovery of the patient's physical function, and according to the patient's psychosis to provide homologous psychological treatment. At the same time, hospice care also includes the care and comfort to the family members of patients, and makes medical staff and family members of patients cooperate with each other, to prolong the survival time of patients, so that the patients’ quality of life can be improved effectively, and the physical and psychological health of family members can also be maintained and enhanced.^[14]^

However, there is still dispute about the effect and implementation mode of hospice care at the present stage, so through the method of systematic evaluation, we compared the effects of hospice care and routine nursing mode on the quality of life and negative emotion of advanced tumor patients, and to promote the quality of life of advanced tumor patients, and reduce the negative emotion of advanced tumor patients, and provide evidence-based medicine for better implementation of targeted service of hospice care.

## 2 Methods

### 2.1 Protocol register

This research has been registered in advance on the OSF platform, The URL of the registered platform is https://osf.io/registries and the registration number is 47enh.

### 2.2 Ethics and dissemination

All data in this study are from published academic papers, so clinical approval is not suitable for this study.

### 2.3 Eligibility criteria

#### 2.3.1 Types of studies

A randomized controlled trial concerning the influence of hospice care on the quality of life and negative emotions of patients with advanced cancer was carried out without concerning if the study adopting blind method or assignment concealment and the language is limited to Chinese and English.

#### 2.3.2 Types of participants

All the patients in the clinical trial were diagnosed as advanced cancer by clinical manifestations, imaging examination and histopathological examination without mental disorder and cognitive dysfunction. The types of tumor include lung cancer, gastric cancer, colon cancer, liver cancer, etc, with the expected survival time less than or equal to 6 months.

Inclusion criteria: randomized controlled trial (RCT); All patients were diagnosed as advanced cancer by clinical manifestations, imaging examination and histopathological examination, and the clinical diagnosis met the diagnostic criteria of all kinds of malignant tumors. All patients were advanced cancer patients who met the receiving standard of hospice care department and received hospice care services; At the time of admission, the estimated survival time should be more than or equal to 1 month but less than or equal to 6 months, and the age should be more than 18 years old; The patients who should be of clear consciousness, and can accurately express their own feelings and needs without previous history of mental illness or mental retardation; The patients who receive the informed consent and take part in the quality of life assessment of patients with advanced cancer voluntarily.

Exclusion criteria: Patients receive hospice care service but the actual life span is less than 1 month; During the period of study, patients have vague consciousness, coma, delirium, and mental symptoms so that they are unable to express their feelings and needs clearly; Patients still actively request and receive radiotherapy and chemotherapy, or patients have targeted treatment; Patients or their families refuse to participate in the investigation during the study; Review and basic research; Patients with incomplete literature of descriptive research and data report.

#### 2.3.3 Types of interventions

Control group: routine basic nursing (including testing patients’ vital signs, guiding patients to use drugs reasonably, ward environment, pain nursing, and skin nursing, etc).

Experimental group: Hospice care + routine basic care.

Hospice care includes: Psychological nursing intervention: Patients with advanced cancer have lost the hope of life, and their mood fluctuates greatly. Nurses should establish a good communication relationship when they are conscious, with mild tone and cordial attitude. The patients should be given enough patience to divert their attention, and to keep patients an optimistic and calm heart. At the same time, patients’ families and friends should be called up to visit patients frequently, and to play soft music for patients to relax their body and mind; Skin care intervention: patients who cannot take care of themselves should be cleaned regularly to keep their skin clean and comfortable; Pain nursing intervention: according to the pain relief principle of 3 steps stipulated by WHO, appropriate pain relief measures should be taken for patients; Diet nursing intervention: nurses should give patients food with high protein, high calorie, rich vitamin, and easy digestion to provide nutrition for the patients. If necessary, nutrition supports such as nasal feeding or jejunum can be used; Environmental care: disinfection and cleaning should be carried out regularly for inpatient wards and windows should be open regularly to ensure the air circulation in the wards. Besides, quiet and tidy environment of the wards should be guaranteed to provide patients with a more comfortable environment and atmosphere.

#### 2.3.4 Types of outcomes

The primary outcome indicators:

1. Overall life quality score;^[15]^
2. Self-rating anxiety scale;
3. Self-rating depression scale.

The secondary outcome indicators:

1. Physical function score;
2. Cognitive function score;
3. Emotional function score;
4. Social function score;
5. Material life score;
6. Nursing satisfaction.

### 2.4 Search methods for identification of studies

Computer retrieval network electronic database: Retrieval CNKI, Chinese clinical trial registry, WANFANG database, China Biology Medicine disc, CQVIP database, PubMed, Embase, The Cochrane Library, and Web of Science database on the clinical research of hospice care on the quality of life and negative emotions of advanced tumor patients. At the same time, the references included in the research were traced and relevant abstracts, papers, and relevant magazines without electronic edition were searched manually. The range of retrieval time was from the beginning of every database establishment to April 1, 2020 by adopting the combination method of theme words and free words. The key words of Chinese search are: “Lin Zhong Guan Huai” or “An Ning Liao Hu” or “Gu Xi Hu Li” or “Zhong Mo Qi Guan Huai” or “Lin Zhong Hu Li” and “Zhong Liu Wan Qi” or “Ai Zheng Wan Qi” and “Sheng Huo Zhi Liang” or “Fu Xing Qing Xu” or “Sui Ji Dui Zhao Shi Yan.” English search terms are: “hospital care” or “palliative care” or “hospice care” or “end of life care” and “advanced tumor” or “advanced cancer” or “neoplasses” and “quality of life” or “negative impressions” and “random controlled trial.” The retrieval of PubMed database was taken as an example, and see Table 1 for retrieval strategy.

**Table 1:**
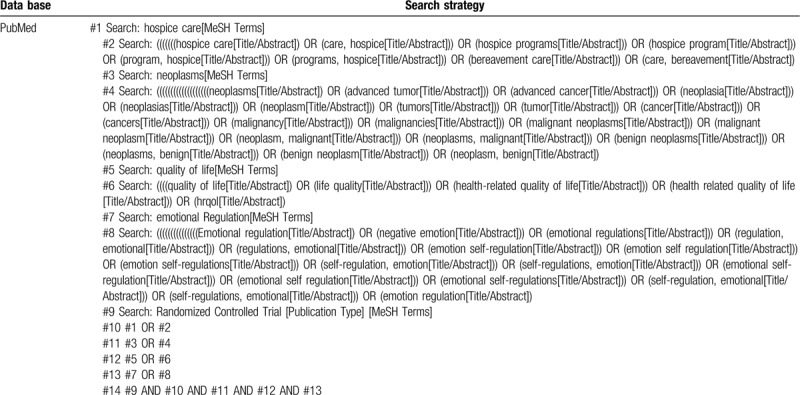
Search strategy used in PubMed database.

### 2.5 Data collection

#### 2.5.1 Study selection

**Figure 1:**
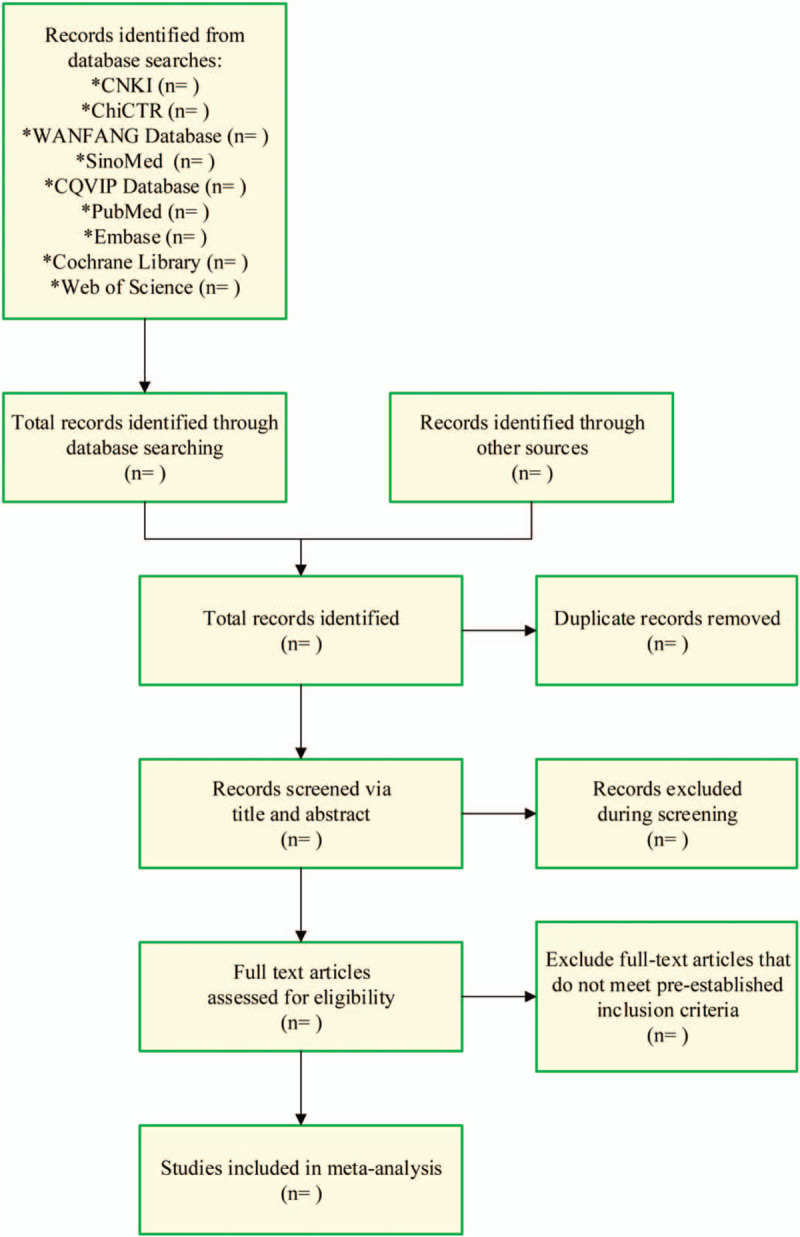
Flowchart of literature selection.

Literature Screening: according to the predetermined inclusion and exclusion criteria, the above-mentioned retrieval strategies are used to retrieve relevant Chinese and English literature. The specific screening steps are as follows:

1. Endnote X6 should be used to establish a personal literature database. After removing the repeated literature, all topics and abstracts included in the literature should be read. The irrelevant literature should be excluded according to the above inclusion and exclusion criteria to eliminate the irrelevant literature.
2. We should try our best to collect relevant literature in an all-round way, and the search results should not be affected by the first authors, publishing units, and publications. The titles and abstracts of the article should be browsed carefully. By carefully reading the full text of the articles that may be relevant, the documents that may meet the standards should be included to make the final evaluation and screening, excluding case control, summary, animal research, and short review.
3. For the phenomenon of repeated publication or data overlap, the recent published data or the data with large sample shall be used.
4. To avoid bias of literature selection and document quality evaluation, the selection of literature in this study is conducted by 2 reviewers independently. In case of divergence, the third researcher will be invited to join in and decide whether to include it after discussion. See Fig. 1 for the flow chart of literature screening. The literature screening process is shown in Fig. 1.

#### 2.5.2 Data extraction

According to the data extraction table designed in advance, 2 evaluators extract the data finally included in the literature respectively, and then make cross-checking. In case of any inconsistency, the opinions of the third party shall be solicited and the decision shall be made through joint discussion. The content of data extraction mainly includes: general information such as title, author, publication period, baseline situation of each research subject, experimental design scheme, sample size, intervention measures and established outcome indicators, etc.

### 2.6 Assessment of risk of bias for included studies

The quality of the final included documents will be evaluated by 2 evaluators according to the Cochrane system evaluator's manual, and then the evaluation results will be cross-checked. In case of any inconsistency, the opinions of the third party shall be solicited and the decision shall be made through joint discussion. The content of quality evaluation mainly includes 5 aspects: random method, assignment concealment, blind method, integrity of result data and selective report bias, and each of which is divided into 3 levels:^[16]^

1. Correctness: that is, perfect and sufficient, indicating that the risk of bias is low;
2. Incorrectness: that is, incomplete and inadequate, indicating that there is a high risk of bias;
3. Unclearness: it indicates that there is no relevant information in the original study, or the risk of bias cannot be determined.

### 2.7 Statistical method

#### 2.7.1 Statistical analysis

Statistical analysis was carried out on the relevant data extracted from the final included literature by Using Stata 13.0 statistical software. Standardized mean difference was used as the effect index of this study, and odds ratio (OR) was used as the effect index of this study. 95% of confidence intervals (CIs) is the statistical analysis of the treatment effect. The results are represented by “forest map.” If the range of 95% CIs is over the invalid line of “forest map,” it shows that there is no statistical difference between the test group and the control group; If the range of 95% CIs is located on the side of invalid line of the “forest map,” it indicates that there is statistical difference between the test group and the control group. When 95% CIs of the positive results is on the right side of the invalid line, or 95% CIs of the bad results is on the left side of the invalid line, it indicates that the results are good for the test group; otherwise, the results are good for the control group.

#### 2.7.2 Heterogeneity test

The core calculation of meta-analysis is to combine the relevant statistics in the same or similar multiple studies. According to the principle of statistics, only the studies with homogeneity can be combined with statistics, so the heterogeneity test is required. Tests for heterogeneity, also known as tests for homogeneity, are to test whether there is heterogeneity between independent studies by hypothesis testing. I^2^ is an indicator to measure the heterogeneity, which is generally considered to be not more than 50% in Cochrane system evaluation. If *P* value is more than or equal to .05 and I^2^ value is less than or equal to 50% in the heterogeneity test, it is considered that there is homogeneity among multiple studies, so fixed-effect model will be used to calculate the combined effects. If *P* value is less than .05 and I^2^ value is more than 50%, it is considered that there is heterogeneity among multiple studies. First, it is necessary to analyze the causes of heterogeneity, mainly making the analysis for the heterogeneity source of each research object, research design method, intervention measures, and outcome indicators, etc in each research, and then dealing with them. If it still cannot be eliminated, random effects model shall be used to calculate the combined effect amount. The model takes the variation of each study into account, and corrects the importance of each study to eliminate the influence of heterogeneity among studies. However, it should be noted that the results calculated by using the random effect model should be carefully treated in the interpretation.

### 2.8 Sensitivity analysis

Sensitivity analysis can be used not only to locate the source of heterogeneity, but also to judge the stability of results. In this study, sensitivity analysis is used to determine the stability of the results. In RevMan 5.3.3 software, sensitivity analysis was carried out according to data characteristics and evaluation results of literature quality. One study should be deleted each time, and a new meta-analysis will be carried out to observe whether the total effect amount (that is, the OR value) and 95% confidence interval (95% CIs) before and after exclusion changed statistically, so as to test the stability of the results. If the result changes greatly after excluding a certain study, it shows the stability of the result is poor, and vice versa.^[17]^

### 2.9 Subgroup analysis

The purpose of subgroup analysis of the included studies is to explore the source of heterogeneity. The criteria of subgroup analysis usually include age, gender, intervention mode, dose size, and treatment time, etc. In this study, we will start with the factors that may cause publication bias, such as the expected survival period, tumor type, whether to take radiotherapy or chemotherapy, etc. and then we will conduct subgroup analysis on the outcome indicators with greater heterogeneity according to the actual situation.

### 2.10 Publication bias

The funnel chart was made in RevMan 5.3.3, and the publication bias was tested by Egger and Begg methods in Stata 13.0 software. The funnel chart shall be observed by taking the merged OR value as the center line to observe whether the 2 sides are symmetrical. In an ideal condition, each study should be evenly distributed on both sides of the vertical line. If there is a bias, the funnel diagram shows a lack of angle, which is shown as the asymmetry of both sides of the vertical line. Funnel chart is to judge whether there is publication bias through visual observation. Because of its strong subjectivity, Egger and Begg methods are used to test funnel chart objectively. When *P* value is more than .05 or 95% CIs including 0 in the result, it shows that there is no publication bias; otherwise, there is publication bias.^[18,19]^

## 3 Discussion

With the development of the times and the progress of human society, the concept of hospice care is more and more popular. It rises with the evolution of medical model and is the product of human understanding of medical essence. Different from the goals of “treatment” and “healing” of traditional medicine, it does not aim at prolonging the survival time of patients, but pays more attention to the quality of life of patients.^[20]^ Hospice care is a rising medical behavior to control and relieve the patients’ physical symptoms and psychological problems at the end of their life when the disease cannot be cured, to provide their families with full support of spirit and society, and finally to improve the quality of life at the end of their life, to maintain and support their physical and mental health, so that the patients can go through the final journey of life calmly, comfortably, and peacefully.^[21,22]^ For patients with advanced cancer, in addition to their physical pain, they also suffer from great psychological pain. Therefore, the application of hospice care to patients with advanced cancer has a positive impact on improving the quality of life and psychological status of patients. However, there are certain controversies about the role of hospice care, personalized real-time strategy, and public recognition. Therefore, this study evaluates the difference between hospice care and traditional nursing through the method of systematic evaluation, and concludes whether hospice care has advantages in improving the quality of life and negative emotions of patients with advanced cancer compared with conventional nursing mode, and the method of making the strategy of personalized hospice care, which provides the basis for the application of hospice care in the treatment of terminal cancer patients.

## Author contributions

**Conceptualization:** Haiyan Qiu.

**Data curation:** Jing Fu, Yan Zeng.

**Formal analysis:** Yan Zeng.

**Funding acquisition:** Haiyan Qiu.

**Investigation:** Jing Fu, Yan Tan.

**Methodology:** Yan Zeng, Baiyu Fu, Yan Tan, Baiyu Fu.

**Resources:** Jing Fu, Yan Zeng.

**Software:** Yan Tan, Baiyu Fu.

**Writing – original draft:** Jing Fu, Yan Zeng, Yan Tan, Baiyu Fu.

**Writing – review & editing:** Haiyan Qiu.
